# Effect of VEGF on Inflammatory Regulation, Neural Survival, and Functional Improvement in Rats following a Complete Spinal Cord Transection

**DOI:** 10.3389/fncel.2017.00381

**Published:** 2017-11-29

**Authors:** Jing Li, Shuangxi Chen, Zhikai Zhao, Yunhao Luo, Yuhui Hou, Heng Li, Liumin He, Libing Zhou, Wutian Wu

**Affiliations:** ^1^Guangdong-Hongkong-Macau Institute of CNS Regeneration, Ministry of Education CNS Regeneration Collaborative Joint Laboratory, Jinan University, Guangzhou, China; ^2^Department of Anatomy, Institute of Basic Medical Sciences, Hubei University of Medicine, Shiyan, China; ^3^Department of Anatomy, University of Hong Kong, Hong Kong, Hong Kong; ^4^Key Laboratory of Biomaterials of Guangdong Higher Education Institutes, Department of Biomedical Engineering, College of Life Science and Technology, Jinan University, Guangzhou, China; ^5^Re-Stem Biotechnology Co., Ltd., Suzhou, China

**Keywords:** spinal cord transection, locomotor function, vascular endothelial growth factor, MAPK signaling, neural circuitry

## Abstract

After complete transection of the thoracic spinal segment, neonatal rats exhibit spontaneous locomotor recovery of hindlimbs, but this recovery is not found in adult rats after similar injury. The potential mechanism related to the difference in recovery of neonatal and adult rats remains unknown. In this study, 342 animals were analyzed. The vascular endothelial growth factor (VEGF) level in spinal segments below injury sites was significantly higher in postnatal day 1 rats (P1) compared with 28-day-old adult rats (P28) following a complete T9 transection. VEGF administration in P28 rats with T9 transection significantly improved the functional recovery; by contrast, treatment with VEGF receptor inhibitors in P1 rats with T9 transection slowed down the spontaneous functional recovery. Results showed more neurons reduced in the lumbar spinal cord and worse local neural network reorganization below injury sites in P28 rats than those in P1 rats. Transynaptic tracing with pseudorabies virus and double immunofluorescence analysis indicated that VEGF treatment in P28 rats alleviated the reduced number of neurons and improved their network reorganization. VEGF inhibition in neonates resulted in high neuronal death rate and deteriorated network reorganization. In *in vivo* studies, T9 transection induced less increase in the number of microglia in the spinal cord in P1 animals than P28 animals. VEGF treatment reduced the increase in microglial cells in P28 animals. VEGF administration in cultured spinal motoneurons prevented lipopolysaccharide (LPS)-induced neuronal death and facilitated neurite growth. Western blots of the samples of lumbar spinal cord after spinal transection and cultured spinal motoneurons showed a lower level of Erk1/2 phosphorylation after the injury or LPS induction compared with that in the control. The phosphorylation level increased after VEGF treatment. In conclusion, VEGF is a critical mediator involved in functional recovery after spinal transection and can be considered a potential target for clinical therapy.

## Introduction

Complete spinal cord injury (SCI) causes devastating neuronal destruction, axonal contact disruption, progressive demyelination and, consequently, complete dissection of the descending motor control pathways below the injury site. Failure of axon regeneration after injury is highly related to the non-permissive local environment in the adult central nervous system (CNS; Mukhopadhyay et al., 1994; GrandPré et al., 2000; Wang et al., 2002; Sivasankaran et al., 2004; Liu et al., 2011; Andrews et al., 2012; Lang et al., 2015). However, several studies have provided evidence that spontaneous regeneration through the lesion site may restore the significant recovery of locomotor function in neonatal rats receiving complete spinal cord transection (ST; Hase et al., 2002a,b; Tillakaratne et al., 2010). For instance, Wakabayashi et al. reported a positive correlation between the number of labeled neurons in each of the supraspinal nuclei and the locomotor performance of the neonatal rats that underwent a complete ST (Wakabayashi et al., 2001). By contrast, some investigators have indicated the lack of signs of regeneration in the transection site by either anterograde or retrograde tracing; this finding indicates that the regaining of some hindlimb functions in neonatal rats may be due to changes in the lumbosacral neuronal circuitry after complete ST, rather than the regeneration of axons across the lesion (Bernstein et al., 1981; Tillakaratne et al., 2010; Yuan et al., 2013). In addition, studies on retransection have provided functional evidence for the shortage of regeneration in the lesion site after neonatal ST, and the lumbosacral neuronal circuitry is confirmed as the structure of functional restoration (Yuan et al., 2013). Furthermore, neuronal death at the primary lesion site and in remote regions is one of main contributors to neurological deficits after SCI (Bisicchia et al., 2016). Thus, it is necessary to promote neuronal survival and significant functional recovery after SCI.

Studies on human and animal models have revealed that functional recovery can be spontaneously regained in SCI due to neuronal survival and intrinsic plasticity in the spinal circuitries that control hindlimb locomotor activity below the lesion (Tillakaratne et al., 2010; Willerslev-Olsen et al., 2015). An increasingly lines of evidences have indicated that the expression levels of cleaved caspase-3 and the proportion of TUNEL-positive cells in the spinal cord are reduced; moreover, motor neuronal survival in the spinal cord ventral horn is enhanced and the locomotor function of rats is improved after SCI (Chen T. et al., 2017; Shen et al., 2017). The RhoA signal may be a significant therapeutic target for enhancing neuronal survival, promoting axon regeneration and improving functional recovery after SCI (Hu and Selzer, 2017). Engraftment of hESC expressing BDNF and GDNF significantly rescues avulsed motoneurons, preserves synaptic covering and reduces astroglial reactivity at the lesion site in rats (Araújo et al., 2017). In the Zebrafish embryo, neuronal survival and growth are influenced by trophic factors (such as insulin-like growth factor-1), which could be attributed to the PI3 Kinase/Akt signaling pathway (Chen S. et al., 2017). In addition, m-TOR and MAPK signals participate in neuronal survival and functional recovery after SCI (Zhou et al., 2016). Therefore, mechanisms underlying neuronal survival within the lumbar spinal cord and locomotor recovery of hindlimbs are very complex after complete ST. In comparison with that in developing CNS, which possesses a transient local micro-environment, nerve regeneration in response to injury is more difficult in adult CNS because of its difficulty in rebuilding an appropriate local microenvironment (Hwang et al., 2014; Qi et al., 2014; Zhang et al., 2014; Keefe et al., 2017). Some differential signaling molecules between the neonatal and mature local microenvironments after ST may be the critical factors that promote the recovery of motor function for repairing adult neural cells.

Vascular endothelial growth factor (VEGF) is a mitogen-activated protein that participates in the formation of new blood vessels and in cellular processes, such as proliferation, growth, and survival. However, the role of VEGF in neural survival and repair remains poorly understood. VEGF is important for neurogenesis and neuroprotection in the pathogenesis of stroke, Alzheimer's disease and motoneuron diseases (Greenberg and Jin, 2005). The enhanced expression of VEGF participates in the VEGF–Nrp1 signaling axis and improves motor function in CMT2D mice; CMT2D mutations alter the conformation of GlyRS, enable GlyRS^CMT2D^ to bind to the neuropilin1 (Nrp1) receptor, and competitively interferes with the binding of the cognate ligand VEGF to Nrp1 (He et al., 2016). In a previous work, VEGF increased the BBB score, reduced the loss of motoneurons and down-regulated the expression levels of IL-1β, TNF-α, and IL-10 in rats that underwent SCI (Wang et al., 2015). Several signaling pathways, such as PI3K/Akt, Erk/MAPK, VEGF/SphK, JNK, and p38 MAPK pathways may be involved in the neuroprotective and nerve regenerative processes of VEGF (Khaibullina et al., 2004; Benedito et al., 2012; Lee et al., 2014; Bhuiyan et al., 2015).

In this study, we propose that VEGF is a key mediator in rats after complete ST and leads to different recovery levels in neonates and adults by regulating inflammatory responses, protecting damaged neurons, and promoting reestablishment of spinal neural circuits.

## Materials and methods

### Animals

All surgical procedures for rats were approved by the Guide for the Care and Use of Laboratory Animals of the National Institutes of Health. Experimental protocols were performed according to the Laboratory Animal Ethics Committee of Jinan University (Permit Number: 20111008001). P1 and P28 female Sprague Dawley rats were supplied by Guangdong Medical Laboratory Animal Center and used in this study (*n* = 342). All animals were kept on a 12/12 h light/dark cycle and given *ad libitum* access to food and water. The animal holding areas constantly monitored, and the temperature and humidity were maintained at 21 ± 2°C and at 44 ± 2 pw, respectively. Anesthetic procedures were used to ensure that animals would not suffer unduly during and after the experimental procedures.

### Surgical procedures for complete ST

P1 rats were anesthetized under deep hypothermia, and P28 rats were anesthetized with 1% pentobarbital sodium (40 mg/kg, i.p.) by using previously described methods (Yuan et al., 2006, 2013; Zeng et al., 2015). A posterior mid-line incision was made to expose the lower thoracic spine. Laminectomy was then performed to expose the T9 spinal cord, and the dura was cut vertically with a pair of microforceps and microscissors. A 2 mm-long segment of the spinal cord at the T9 level was completely removed using angled microscissors (this procedure was not applied in the sham groups). The cut ends of the cord were lifted to confirm the completion of transection and the success of the SCI experimental procedures. The surgical incisions were sutured. Post-surgical animals were placed on a heating pad at 37°C to facilitate their recovery from anesthesia and surgery. After recovery from surgery, P1 pups were housed with their mothers, and adult animals were housed in individual cages. The adult animals then received postoperative cares, including intramuscular injection of penicillin (50,000 U/kg/d) for 3 d and manual emiction two or three times daily until reestablishment of the automatic micturition function. These steps were not required in surgical P1 pups and sham-operated animals. All animals were monitored and studied for up to 2 months after the surgery. All procedures were approved by the Jinan University of Science and Technology's Center of Institutional Animal Care and Use Committee.

### Recombinant VEGF

Recombinant VEGF (CYT-241; ProSpec-Tany TechnoGene Ltd., Ness Ziona, Israel) was dissolved in 0.01 M phosphate buffered saline (PBS) at pH 7.4 according to the manufacturer's instructions. VEGF was used for cell culture and animal experiments.

### Treatment and grouping

P1 rats were randomly assigned to three groups: (1) sham control (only laminectomy), (2) complete ST + PBS, and (3) complete ST + inhibitor (10 nM S3, S3012). The P1 + inhibitor group were injected intraperitoneally with 10 nM pazopanib once daily for 7 d after injury. P28 rats were randomly divided into three groups: (1) sham control (only laminectomy), (2) complete ST + PBS, and (3) complete ST + 0.4 nM VEGF (Herrera et al., 2009). The treatment groups were injected intraperitoneally with recombinant VEGF once daily for 7 d after the surgery. The postoperative survival period was 2 months. The numbers of animals used in each experimental set are summarized in Supplementary Table 1.

### Cytokine and chemokine analysis

P1 and P28 surgical rats were killed with pentobarbital at indicated time points [0 (before the surgery), 6, 12, 24 h, 3, 5, and 7 d after the surgery]. Six animals were used in each time point. The T8 and T10 segments of the spinal cord were rapidly extracted, flash-frozen in liquid nitrogen, and stored at −80°C. The tissues were homogenized on ice supplemented with a protease inhibitor (Complete Protease Inhibitor Cocktail; Santa Cruz Biotechnology, Santa Cruz, CA, USA). Protein fractions were processed using the Ambion PARIS kit according to the manufacturer's instructions and stored at −80°C. Protein concentration was determined by Microplate Reader (Thermo Scientific). Protein samples (2 mg/ml) were analyzed using the Bio-Plex Pro Rat Cytokine 24-plex Panel (171K1001M, Bio-Rad) on a Bio-Plex 200 System and Bio-Plex Pro II Wash Station (Bio-Rad Laboratories) according to the manufacturer's instructions.

### Assessment of locomotor function

At 8 weeks after the surgery and treatments, behavioral tests were performed by the experimenters blinded to the treatments.

#### BBB scale and grid climbing test

Behavioral tests were performed as previously described (Basso et al., 1995). Hindlimb locomotor function was analyzed using the BBB locomotor rating scale (Scheff et al., 2002) and inclined grid climbing assessment (*n* = 12 for each group). For the BBB scoring, SCI rats were placed in an open field (70 × 70 × 50 cm) and allowed to move freely.

In the grid climbing test, animals needed to move from the bottom to the top of a grid placed at 45° from the horizontal plane. This test was designed to monitor the hindlimb capability of spontaneously placing reflex (Ramón-Cueto et al., 2000). The coordination of the forelimbs and hindlimbs was required to fulfill the task. Videos were recorded for more than 5 min to observe the knee joint angle of animals climbing the grid and the number of animals whose hindlimb gripped the grid during climbing. Recording was performed by two investigators who were blinded to the treatments (*n* = 12 for each group). Neither pre-injury training nor food award was employed.

#### Electrophysiology

After the behavioral experiment, spinal motor-evoked potentials (SMEPs) were recorded as previously described (Guo et al., 2007) to evaluate the electrical conduction of motor axons from the lumbosacral spinal cord segment to triceps surae (*n* = 6 for each group). After deep anesthesia (10 mg/kg propofol and 40 mg/kg pentobarbital sodium), the L3-L4 spinal cord and triceps were exposed. The electrodes of the evoked potential recorder (VikingQuest, Nicolet, US) were placed in the L3-L4 spinal cord and triceps surae. A stimulating electrode was placed on the L3-L4 spinal cord, and a recording electrode was inserted in the center of the triceps surae, with a grounding electrode in the subcutaneous tissue. Stimulations (5 mV, 2 ms, 1 Hz) were applied for 20 ms in all animals. The amplitude and latency of the SMEPs were calibrated first and then measured according to the standardized protocols (Guo et al., 2007; Alam et al., 2017).

### Pseudorabies virus (PRV) tracing

Retrograde labeling was performed using PRV-152 containing the CMV-EGFP reporter gene (provided by Gary Pickard). The viral recombinants were amplified according to previously established protocols (Han et al., 2015). At 8 weeks post-injury, the animals were anesthetized with 1% pentobarbital sodium, and the right triceps surae were exposed. PRV-152 (6 μl) was injected at three different sites by using a 10 μl Hamilton syringe fitted with a 33-gauge needle (**Figure 5A**). At 72 h after the labeling, the animals were killed by overdose injection of 1% pentobarbital sodium, followed by subsequent transcardial perfusion with 0.9% saline and 4% cold paraformaldehyde in 0.1 mol/l PBS (pH 7.4). The L3 spinal cord segment was dissected and collected. Frozen sections with a thickness of 30 μm were cut. GFP signal was detected by a fluorescence microscope (Zeiss LSM700).

### Tissue processing

At 8 weeks after the surgery, the animals were euthanized by deep anesthesia with 1% pentobarbital sodium (50 mg/kg, i.p.) and transcardially perfused with saline followed by 4% paraformaldehyde (PFA) in 0.1 M PBS (pH 7.4). The L3 segment of the spinal cord was dissected and post-fixed in 4% PFA for 24 h. The tissue was dehydrated with 15% sucrose at 4°C for 24 h followed by 30% sucrose at 4°C for 24 h or overnight. The samples were embedded in OCT compound and stored at −80°C. Successive sections of the spinal cord were cut transversely at thicknesses of 15 and 20 μm by using a Leica cryotome (CM1950, Germany).

### Histology and immunohistochemistry

Nissl staining (1% neutral red) was performed on 15 μm-thick frozen sections for histological analysis to observe the gross morphology. Immunohistochemistry analysis was carried out on 20 μm frozen sections. The goat anti-choline acetyltransferase (ChAT) antibody (1:500, AB144p, Millipore) was used to characterize spinal motoneurons. Neurons in the lumbar enlargement of the spinal cord in rats were labeled with the NeuN anitibody (1:1,000, ab177487, Abcam). The neurites of primary spinal motoneurons were stained with the Map-2 antibody (1:500, ab5392, Abcam) in rats. The Iba1 antibody (1:500, ab5076, Abcam) was used for microglial staining to study inflammation after injury. All signals were detected through fluorescence analysis using secondary antibodies conjugated to Alexa Fluor 546 or 488 (1:1,000, Invitrogen).

### Cell counts

To investigate changes of spinal neurons and microglia in the lumbar enlargement, coronal sections from the L3 segment, were prepared 2 months after injury and allocated to three alternative series of 10 sections. The first series was stained with 1% neutral red (Nissl staining), the second one with anit-NeuN and anti-ChAT, and the third one with Ibal-1. Motoneurons were identified by the large soma (with diameter larger than 30 μm) with a clear nucleus and Nissl bodies by using a previously described method (Li et al., 1994). The number of Nissl-positive motoneurons (with diameter larger than 30 μm) and interneurons (10–30 μm in diameter) was estimated on two sides of each section in the ventral horn. The mean from one series of section was regarded as one sample and calculated using Image J 5.0 software. Six animals were used in each group. ChAT-positive cells were classified into two types: motor neurons located in the ventral horn with large cell bodies, and interneurons located around the central canal. Spinal motoneurons in the L3 segment, large NeuN-positive and ChAT-positive neurons were counted in both ventral horns. The mean from one series of section was taken as one sample. Six animals were used in each group. The number of Ibal-1-positive microglia was calculated on two sides of each section in the ventral horn.

To analyze the PRV-labeled cells in the L3 segment, sections were divided into six alternative series of 10 sections. We counted cells positive for the tracer on the right side in every section by Image J 5.0. The mean from six series of 10 sections was taken as one sample. Six animals were used in each group.

### Primary culture and treatment of rat spinal motoneurons

E15 rat embryos (*n* = 18) were used to obtain spinal motoneurons by using a previously described method (Leach et al., 2011). Finely separated motoneurons were seeded in a volume of 200 μl at the following densities per well: 2 × 10^3^ cells in 96-well plates, 5 × 10^3^ cells in 8-well plates, and 1 × 10^5^ cells in 24-well cell culture plates. The plates were pre-coated with 100 μg/ml poly-D-lysine (PDL) (Beyotime Institute of Biotechnology, Shanghai, China). The motoneurons were cultured in DMEM/F-12 culture medium (HyClone™; Thermo Fisher Scientific, Inc.) supplemented with 10% fetal bovine serum (FBS) (Sijiqing Biotech Corp) and 1% penicillin/streptomycin (P/S) (Solarbio Biotech Corp) for 4 h to enable the cells to adhere to the plates. The medium was aspirated and replaced with Neurobasal-A (Life Technologies) culture medium supplemented with 2% B-27 (Life Technologies) and 1% P/S.

After 24-h incubation, the primary motoneurons were pre-treated with 0.4 nM recombinant VEGF and inhibitors for 2 h and coincubated with 1 μg/ml lipopolysaccharide (LPS; a neuroinflammation inducer) for 24 h (Herrera et al., 2009; Sanchez et al., 2010; Seiler et al., 2017; Xu et al., 2017). After the indicated time points, the neurons were used to perform MTT assay or fixed with 4% PFA for immunofluorescence staining. Whole-cell lysates were collected for Western blot analysis.

### Western blot analysis

The whole-cell lysates from primary motoneurons and tissue lysates from T8, T10, and L1-5 segmental samples in rats were collected in a RIPA buffer mixture (Solarbio Biotech) supplemented with PMSF (1:100; Solarbio Biotech), mixed with 20% sample loading buffer, and heated for 15 min at 95°C. Protein samples were resolved by 10% sodium dodecyl sulfate-polyacrylamide gel electrophoresis (SDS–PAGE) and transferred onto polyvinylidene difluoride (PVDF) membranes (Millipore, Billerica, MA). After blocking in 5% bovine serum albumin (BSA) in Tris–HCl buffer supplemented with 0.1% Tween-20 (TBST, pH 7.4) for 1 h, the membranes were incubated with the following specific antibodies at 4°C overnight: mouse anti-VEGF (1:1,000, MABC595, Millipore), rabbit anti-pErk1/2 (1:1,000, ab4370, Abcam), rabbit anti-Erk1/2 (1:1000, ab4695, Abcam), and mouse anti-GADPH (1:2,000, sc-365062, Santa Cruz Biotechnology). After washing the membrane with 0.1% TBST three times for 5 min each at room temperature (RT), horseradish peroxidase-conjugated goat anti-mouse secondary antibodies (1:1,000, Boster) or goat anti-rabbit secondary antibodies (1:1,000, Boster) were used. The membranes were washed in 0.1% TBST three times for 5 min each at RT. The immunoreactive bands were visualized using a chemiluminescence (ECL) kit (Bio-Rad Laboratories). Signal intensity was quantified using Image J software.

### Statistical analysis

All statistical analyses were performed using GraphPad Prism 6 software. Data were reported as mean ± standard deviation (*SD*) and analyzed using ANOVA or repeated-measure ANOVA. If equal variances were found, then the least significant difference test was applied; otherwise, Kruskal–Wallis test and Dunnett's T3 test were used. A *p* < 0.05 was considered to indicate statistical significance.

## Results

### Spinal cords from neonates maintain higher VEGF expression than those from adults after complete ST

**Figure 1:**
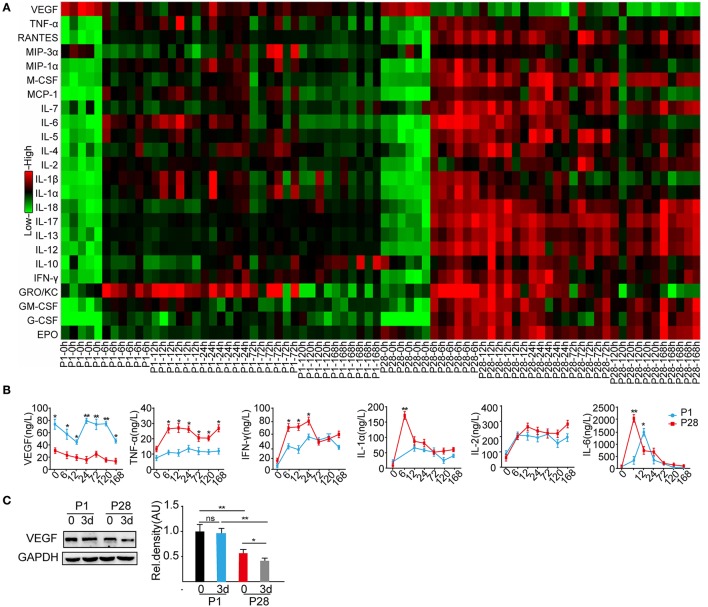
Cytokine profiling of P1 and P28 rats following complete ST. **(A)** Bio-Plex Pro Rat Cytokine 24-plex Assay Heatmap showed the expression of 24 cytokines in the spinal cords of P1 and P28 rats at different timelines after injury; the color from green to red presents the concentration of cytokines from low to high; **(B)** Changes in the protein expression of representative cytokines, such as VEGF, TNF-α, IFN-γ, IL-1α, IL-2, and IL-6. VEGF expression significantly changed at every timeline between the two groups (*n* = 6, ^*^P < 0.05, ^**^P < 0.01). The levels of VEGF, TNF-a, IFN-γ, IL-1a, and IL-6 were significantly higher in P1 ST rats than those in P28 ST rats, but the IL-2 protein level was similar between the two groups (*n* = 6, ^*^P < 0.05, ^**^P < 0.01); **(C)** Western blot showed the protein expression of VEGF in the spinal cord of neonatal and adult rats at pre-surgery and 3 days after the surgery. After ST, the VEGF expression was significantly decreased in P28 rats but not in P1 rats compared with the levels before the surgery. Before and after injuries, the VEGF expression was significantly lower in P28 rats than that in P1 rats (*n* = 6,^*^P < 0.05, ^**^P < 0.01).

After SCI, the local microenvironment critically influences neuronal survival, axonal regeneration, and functional recovery (Liu et al., 2011; Zhang et al., 2014; Dulin et al., 2015). As described before, neonate rats behaved spontaneous functional recovery after the complete thoracic segment transection, but this recovery is not found in adult rats after similar injury (Yuan et al., 2013). To search for the possibly discrepant cytokines and inflammatory mediators responding to complete ST in P1 and P28 animals, the Bio-Plex Pro Rat Cytokine 24-plex immunoassay was performed on T8 and T10 segmental samples at 0, 6, 12, 24 h, 3, 5, and 7 d after the T9 complete transection. The VEGF levels were significantly higher in P1 animals than those in P28 animals at all time points after the surgery (Figures 1A,B). The levels of cytokines including TNF-α, IFN-γ, IL-7, IL-12, IL-13, and IL-17 decreased in P1 animals compared with those in P28 animals (Figure 1B and Supplementary Figures 1A,B; *n* = 6, *P* < 0.05 or 0.01). The cytokine IL-10 increased in P1 animals compared with that in P28 animals at post-injured 7 d (Supplementary Figure 1A, *n* = 6, *P* < 0.05). The levels of IL-2 and IL-4 were not significantly different between the two groups (Figure 1B and Supplementary Figure 1A, *n* = 6, *P* < 0.05). At 12 h post injury, the IL-4 level significantly differed between the two groups. Moreover, the IL-6 level peaked at 24 h after injury in neonates and at 6 h in adult rats (Figure 1B, *n* = 6, *P* < 0.05). Considering that cytokines after SCI have been widely studied (Sköld et al., 2000; Wang et al., 2015), here we mainly focused on the role of VEGF after the injury. Western blot analysis of T8 and T10 spinal samples indicated the significantly higher VEGF protein levels in P1 animals than those in P28 animals before surgery. Furthermore, the VEGF level further decreased in P28 animals 3 days after the surgery, but this decrease was not detected in P1 rats (Figure 1C, *n* = 6, *P* < 0.05 or 0.01). Thus, VEGF may be an important mediator responsible for rare functional recovery in adults after complete thoracic segment transection.

### VEGF treatment improves locomotor recovery in rats after complete ST

To further evaluate VEGF function after ST, we treated P1 ST rats with VEGFR inhibitor to block VEGF function and P28 ST animals with the recombinant VEGF, and then observed their hindlimb functional recovery by behavioral tests.

**Figure 2:**
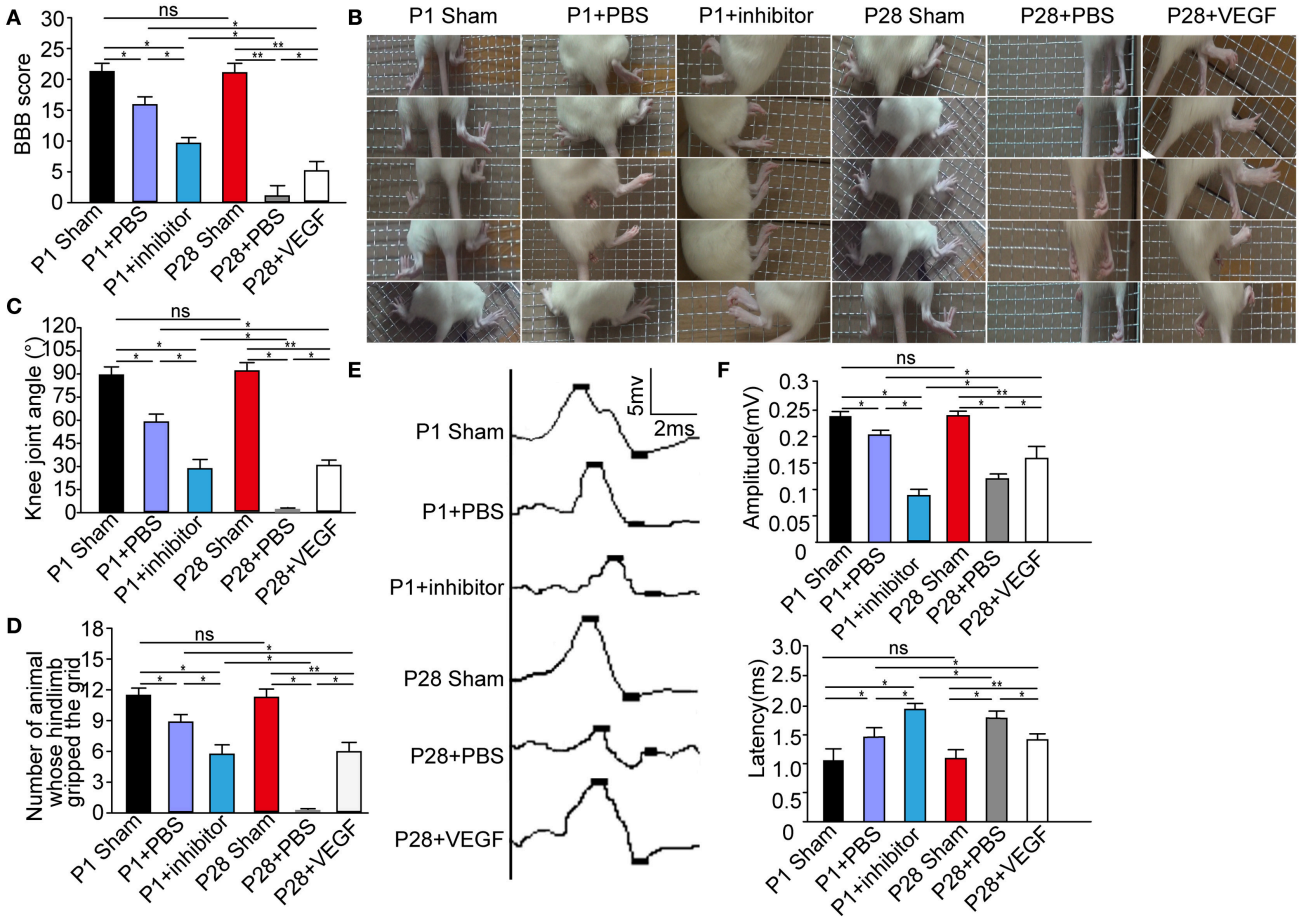
VEGF treatment improves locomotor recovery in rats after complete ST. **(A)** The BBB scores in the P28 + PBS group were significantly lower than those in P1 + PBS, P28 + VEGF, and P28 sham groups; the scores in the P1 + inhibitor group were also significantly lower than those in P1 + PBS and P1 sham groups but higher than those in the P28 + PBS group; **(B)** In grid climbing test, continuous time-lapse images from the video showed alternative movements of two hindlimbs, involving the flexion and extension of the three major joints (the hips, the knees, and the ankles) in sham and P1 + PBS groups but not in the P28 + PBS groups. In P28 + VEGF and P1 + inhibitor group, slight movements of two or three joints were found; **(C)** The knee joint angles were assessed in six groups: the value of knee joint angle was significantly decreased in P1 and P28 ST rats compared with that in sham groups, was higher in the P28 + VEGF group than that in the P28 + PBS group, and was lower in the P1+ inhibitor group than that in the P1 + PBS group; **(D)** The number of animals whose hindlimb gripped the grid was shown in six groups; **(E)** SMEPs were recorded in six groups: P1 sham, P1 + PBS, P1 + inhibitor, P28 sham group, P28 + PBS, and P28 + VEGF groups; **(F)** SMEPs significantly decreased in P1 and P28 ST rats compared with that in sham rats. The peak value of the amplitude significantly reduced in P1 rats with the inhibition of VEGF compared with that in the P1 + PBS group. The peak value of amplitude significantly increased in P28 rats treated with VEGF compared with that in the P28 + PBS group. The changes in the latency showed an opposite trend to that of the amplitude in these groups. ^*^P < 0.05; ^**^P < 0.01; *n* = 12 in each group.

Consistent with previous observations (Yuan et al., 2013), after PBS treatment, P1 ST rats showed evident hindlimb functional recovery (Supplementary Video 1), but such effect was not detected in P28 ST rats (Supplementary Video 2). Interestingly, P1 ST rats subjected to 1 week of postoperative inhibition of 10 nM pazopanib showed the decreased functional recovery at 8 weeks after injury (Supplementary Video 3). P28 ST rats with 1 week of postoperative treatment of VEGF (5 μg/kg) showed slight movement of the hip joint at 2 weeks after injury, subsequently extensive movement of the hip joint and slight movement of the knee joint at 3 weeks after injury, and slight movement of the knee and ankle joints and extensive movement of the hip joint tended to stabilize 6 weeks after the surgery (Supplementary Video 4). As shown in Figure 2A, the Basso, Beattie, and Bresnahan (BBB) scores of P1 and P28 ST animals were significantly lower than those in P1 and P28 sham groups at 2 months after injury. No significantly statistical differences were found between P1 and P28 sham groups. After VEGF administration, the BBB scores significantly increased in P28 ST rats compared with those in the P28 + PBS group, but this increase did not reach the level in the P1 + PBS group (*n* = 12, ^*^P < 0.05, ^**^P < 0.01). On the other hand, inhibition of VEGFR in ST neonates decreased the BBB scores compared with those in the P1 + PBS group, and this decrease did not reach the level of the P28 + PBS group with low VEGF expression (Figure 2A, *n* = 12, ^*^P < 0.05, ^**^P < 0.01). The results of the grid climbing test indicated no significant difference in the knee joint angle of animals climbing the grid between P1 and P28 sham groups (Figures 2B,C, *n* = 12 in each group). After injury, the knee joint angle in P1 ST rats significantly decreased in comparison with that in the P1 sham group. The angle of P28 ST rats significantly reduced in comparison with the P28 sham group (Figure 2C, *n* = 12, ^*^P < 0.05, ^**^P < 0.01). The angle in the P1 + inhibitor group was significantly smaller than that in P1 + PBS and P1 sham groups (30.8° ± 3.76° vs. 58.9° ± 3.65°, 89.7° ± 3.81°, Figure 2C, *n* = 12, ^*^P < 0.05, ^**^P < 0.01). By contrast, the angle in the P28 + VEGF group was significantly larger than that in the P28 + PBS group (31.4° ± 2.84° vs. 0° ± 1.50°), significantly smaller than that in P1 + PBS and P28 sham groups (31.4° ± 2.84° vs. 58.9° ± 3.65° and 90.6° ± 3.52°, Figure 2C, *n* = 12, ^*^P < 0.05, ^**^P < 0.01). As shown in Figure 2D, it was found that compared with P1 and P28 sham groups, the number of animals whose hindlimb gripped the grid during climbing significantly decreased in P1 + PBS, P1 + inhibitor, and P28 + VEGF groups. But in the P28 + VEGF group, the number was significantly more than the P28 + PBS group. After inhibition of VEGF, the number in the P1 + inhibitor group was less than that in the P1 + PBS group (*n* = 12, ^*^P < 0.05, ^**^P < 0.01).

To further assess functional status, we recorded the SMEPs of ST rats 2 months after surgery. We stimulated L3 spinal segment and identified the triceps surae. As a widely applied clinical evaluation, the latency of SMEPs reflects the number of excited axons of the nerve, and the peak of SMEPs corresponds to the conduction velocity of the nerve. The amplitude of SEMPs decreased and the latency increased in neonatal and adult ST rats compared with that in sham groups, but the amplitude was smaller and the latency was longer in the P28 + PBS group compared with those in the P1 + PBS group (Figure 2E). After treatment with VEGF, the peak amplitude significantly increased in P28 ST rats compared with that in the P28 + PBS group, but the recovery did not reach the levels in the P1 + PBS group (Figure 2F, *n* = 12, ^*^P < 0.05, ^**^P < 0.01). Additionally, administration of the inhibitor in neonatal animals significantly decreased the peak amplitude and significantly increased the latency of the SMEPs compared with that in the P1 ST animals, and the difference was significant in contrast to that in the P28 + PBS group (Figure 2E, *n* = 12, ^*^P < 0.05, ^**^P < 0.01).

### VEGF facilitates neuronal survival and local network reorganization below injury sites after ST injury

**Figure 3:**
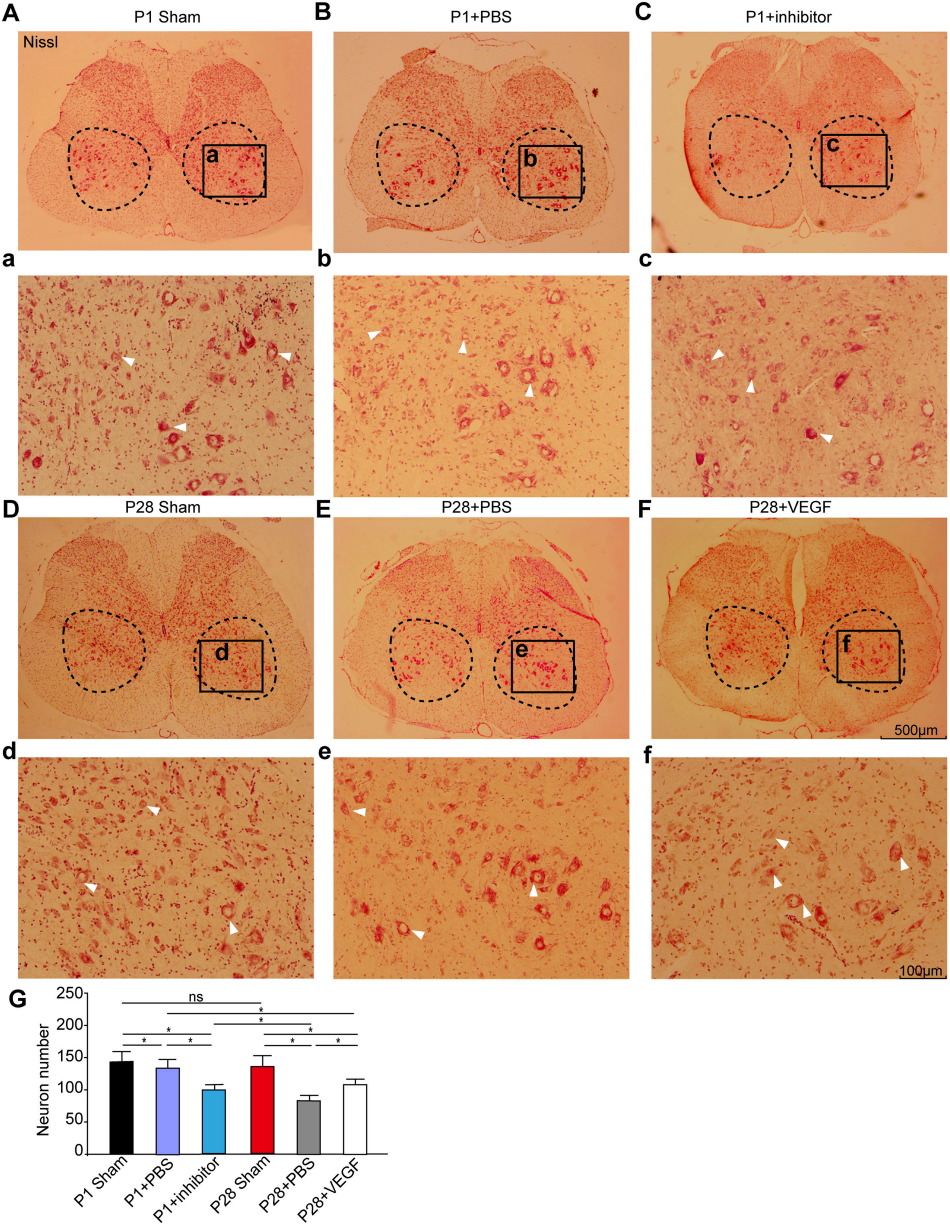
VEGF enhances spinal neuron survival in rats undergoing ST. **(A–F)** Nissl staining was carried out on spinal transverse sections of the L3 segment and high power inserts **(a–f)** in the P1 sham group **(A,a)**, the P1 + PBS group **(B,b)**, the P1 + inhibitor group **(C,c)**, the P28 sham group **(D,d)**, the P28 +PBS group **(E,e)** and the P28 + VEGF group **(F,f)**; **(G)** Statistical analysis showed that the number of motoneurons and interneurons significantly decreased after lesion in both neonatal and adult rats compared with that in the sham groups, was not significantly different between P1 and P28 sham groups, and decreased in the P28 + PBS group compared with that in the P1 + PBS group. Treatment of VEGF in P28 ST animals can significantly reverse the tendency compared with that in the P28 + PBS group, and inhibition of VEGF in P1 ST rats significantly decreased the number in contrast to that in the P1 + PBS group (*n* = 6, ^*^P < 0.05, ^**^P < 0.01). The dashed boxes in **(A–F)** indicate the regions where the number of motoneurons was estimated (>30 μm in diameter, arrows indicated) and interneurons (10–30 μm in diameter, arrows indicated). The black box in **(A–F)** represents the region of high power inserts **(a–f)**.

In our ST model, we completely removed the T9 spinal segment, and hindlimb functional recovery is impossibly attributable to axon growth across the lesion sites. The local neural circuit reorganization below the injury site may be the main reason to explain functional recovery. To test this, we firstly compared neuronal numbers on transverse sections from the L3 segment at 8 weeks after surgery. As shown in Figure 3, Nissl staining labeled all neurons in the gray matter, and large spinal motoneurons and interneurons were visible in the ventral horn. The laminectomy did not influence the number and morphology of spinal motoneurons and some interneurons in the L3 segment in P1 and P28 sham groups. The number of surviving spinal neurons in the L3 segment in both neonatal and adult rats decreased after transection, and the reduction was more prevalent in the P28 + PBS group than that in the P1 + PBS group. After treatment with VEGF, the neuron number in P28 ST animals was significantly higher than that in the P28 + PBS group but was significantly lower than that in P1 ST rats. Additionally, the neuron number in the P1 ST rats with inhibitor significantly decreased compared with that in the P1 + PBS group (Figure 3, *n* = 6, ^*^P < 0.05, ^**^P < 0.01). This finding indicates that VEGF slows down neuronal death after injuries.

**Figure 4:**
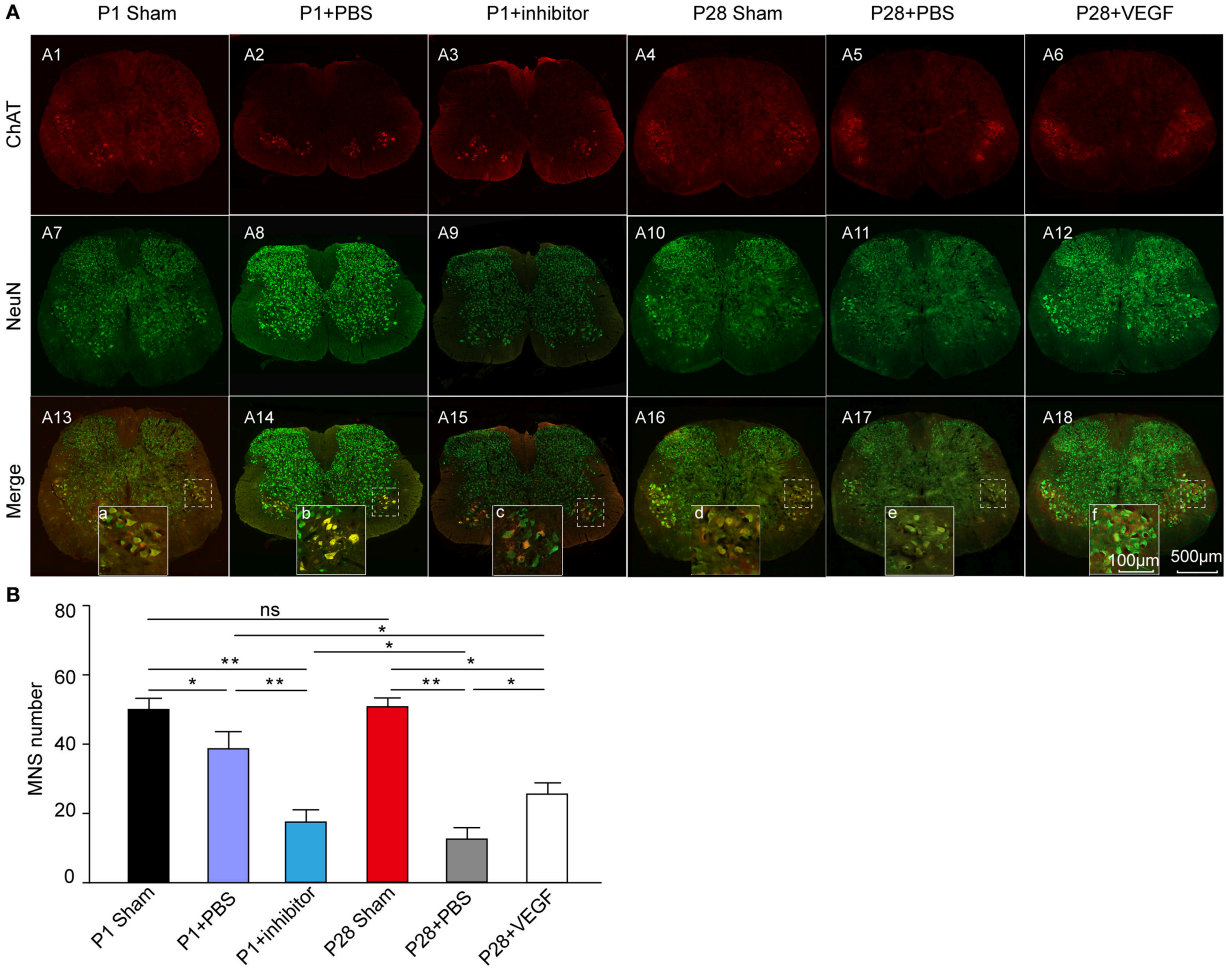
Exploration of the effect of VEGF on spinal motoneuron survival in rats undergoing ST. **(A)** Immunofluorescence staining for ChAT (red, **A1–A6**) and NeuN (green, **A7–A12**) conducted in the spinal transverse section of the L3 segment, and the merged images are presented in **(A13–A18)**; **(B)** Bar graph shows that the number of motor neurons significantly decreased after lesion, and this effect was reversed by VEGF treatment; inhibition of VEGF in P1 rats resulted in high death rate of spinal motor neurons (*n* = 6, ^*^P < 0.05, ^**^P < 0.01). The dotted boxes in **(A13, A14)** indicate the area of high-power inserts **(a–f)**.

Secondly, to specifically analyze changes in spinal motoneurons, we conducted double immunostaining of transverse sections from the L3 segment by using anti-NeuN and anti-ChAT antibodies (Figure 4A). The number of ChAT-positive motoneurons was not significantly different between P1 and P28 sham groups, and significantly decreased in ST neonates and adults compared with that in sham groups. After VEGF treatment, the motoneuron number in P28 ST rats significantly increased compared with that in the P28 + PBS group but lower than that in the P1 ST animals. After VEGF inhibition, the number of motoneurons in P1 ST animals significantly decreased compared with that in the P1 + PBS group (Figure 4B, *n* = 6, ^*^P < 0.05, ^**^P < 0.01).

**Figure 5:**
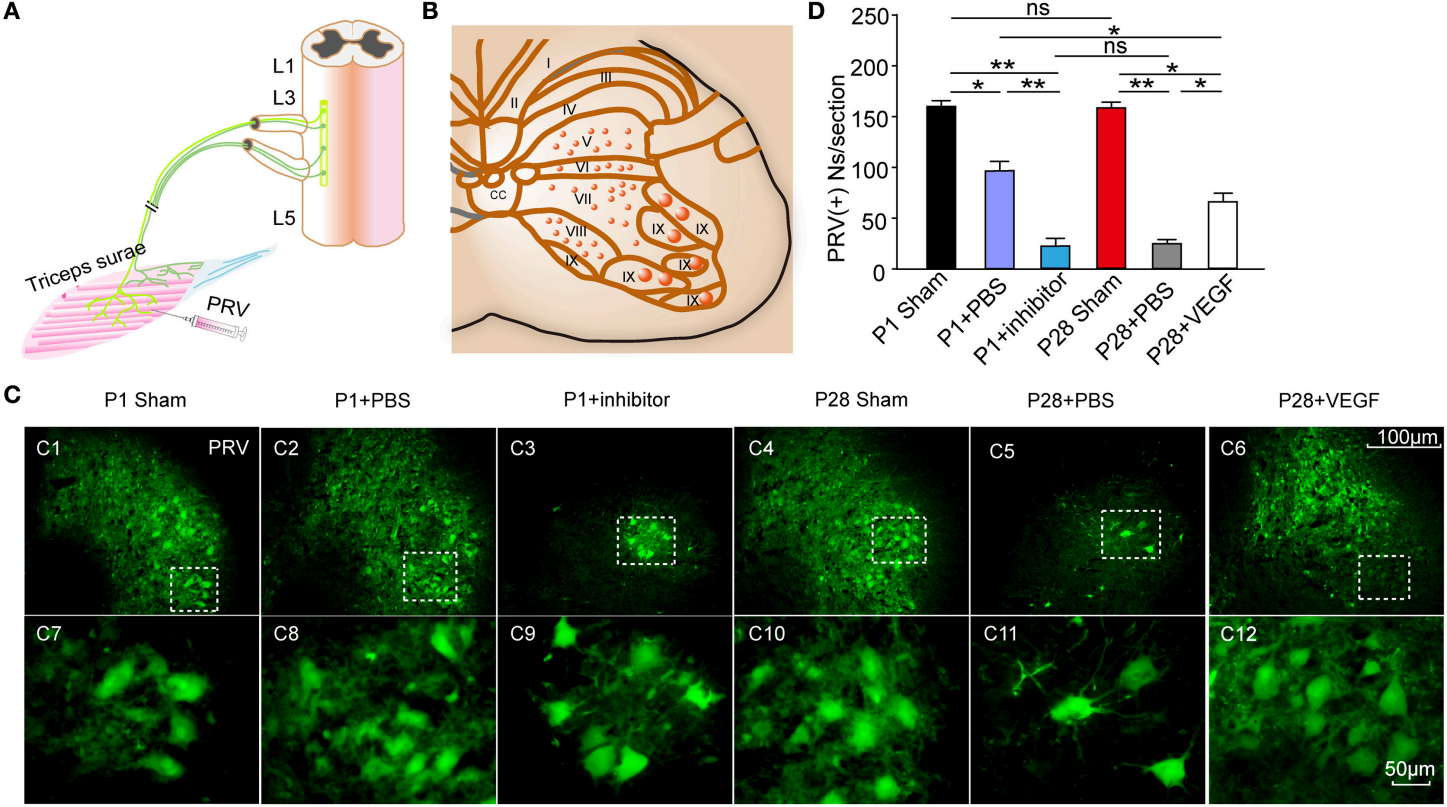
Effect of VEGF on the reconstruction of intrinsic spinal circuitry after ST. **(A)** Schematic illustrating PRV injection in the right triceps to label neurons in the L3 segment; **(B)** Schematic diagram of gray matter lamina in the spinal cord; **(C)** After 72 hr, PRV (green)-labeled spinal neurons in laminae V-VIII on the ipsilateral side **(C1–C6)** and their high-power inserts **(C7–C12)** indicated as dotted boxes in **(C1–C6)**, respectively. **(D)** The number of PRV-labeled neurons significantly decreased after injuries compared with that in sham groups; no significant difference was observed in the number of PRV-labeled neurons between P1 and P28 sham groups; the number of these neurons was higher in the P1 + PBS group than that in the P28 + PBS group; the number in the P28 + VEGF was significantly higher than that in the P28 + PBS group; and the number in the P1 + inhibitor group was significantly lower than that in the P1 + PBS group; ^*^P < 0.05; ^**^P < 0.01; *n* = 6 in each group; *t*-test.

To determine whether or not ST injury induces the reorganization of the spinal local network, we injected the transynaptic pseudorabies virus (PRV) into the triceps surae, and analyzed the number of labeled spinal motoneurons and segmental spinal interneurons that produce synapses with spinal motoneurons, in the L3 segment at 8 weeks post-injury (Figures 5A–C). After 72 h of the inoculation, ipsilateral motoneurons were retrogradely labeled in the ventral horn (lamina IX) and smaller interneurons were retrogradely infected in layers V–VIII in transverse sections of the L3 segment (Figures 5C1–C6). The number of PRV-labeled neurons was significantly lower in the P1 + PBS group than that in the P1 sham group (87.0 ± 7.50 vs. 158. 0 ± 3.52) and in the P28 + PBS group than that in the P28 sham group (19.0 ± 3.02 vs. 156.0 ± 4.26 cells per section; Figure 5D). The number of PRV-labeled neurons was not significantly different between P1 and P28 sham groups (158.0 ± 3.52 vs. 156.0 ± 4.26 cells per section; Figure 5D). The number of PRV-labeled neurons was significantly lower in the P28 + PBS group compared with that in P1 + PBS and P28 + VEGF groups (19.0 ± 3.02 vs. 87.0 ± 7.50, 65.0 ± 2.53, cells per section; Figure 5D, *n* = 6). Moreover, the number of PRV-labeled neurons was significantly lower in the P1 + inhibitor group compared with that in the P1 + PBS group (21.0 ± 4.10 vs. 87.0 ± 7.50, cells per section; Figure 5D, *n* = 6).

These observations indicate that VEGF fosters the reorganization of the spinal motor network after complete ST.

### VEGF prevents inflammation in chronic periods of repair after ST

**Figure 6:**
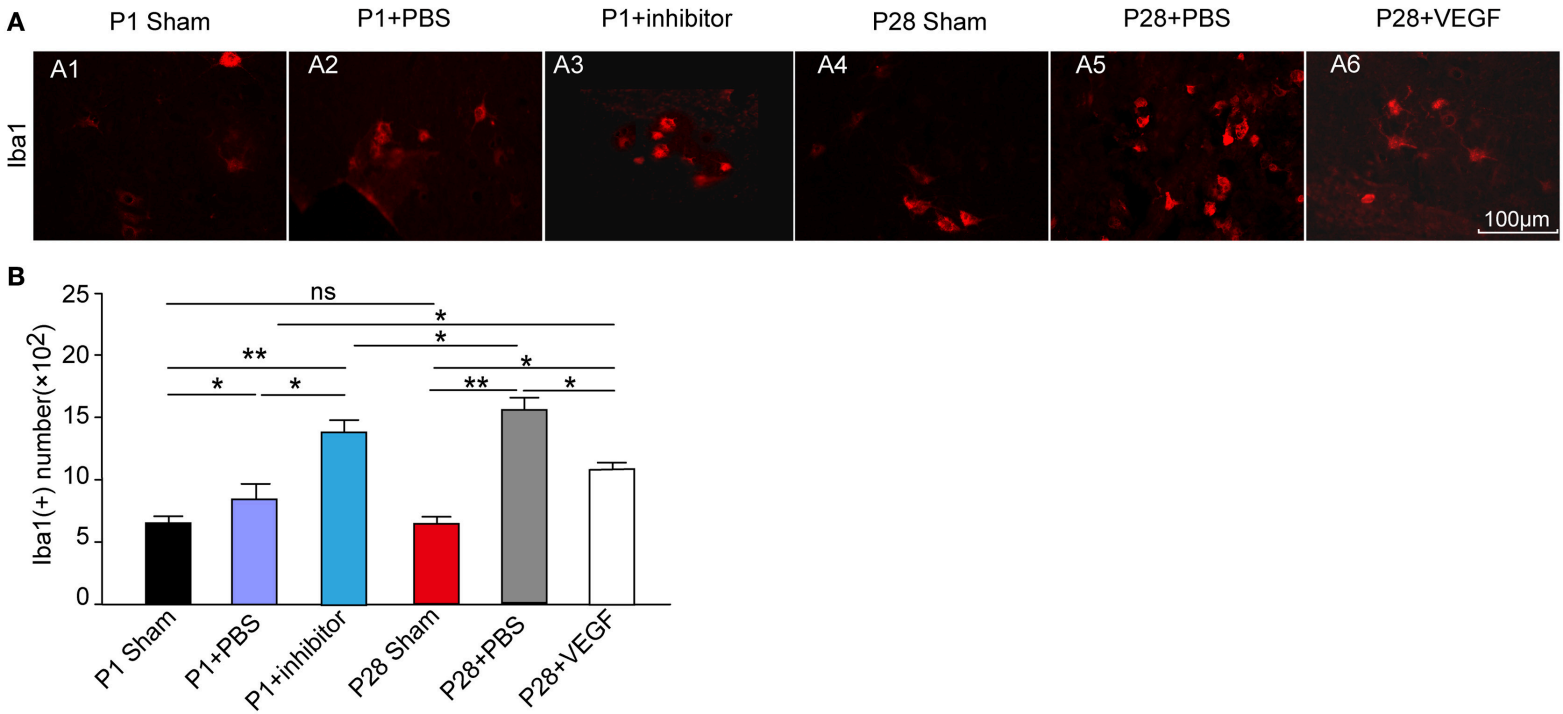
Protective role of VEGF against inflammation in spinal cord in rats after ST. **(A)** Immunofluorescence staining for Ibal-1 to label microglia (red, **A1–A6**) in the L3 segment of the rat spinal cord; **(B)** The number of Ibal 1-positive cells increased in the four injured groups compared with that in the sham groups. There was no significant difference in the Ibal-1 positive cell number between the P1 and P28 sham groups. Among the four injured groups, the number in the P28 + PBS group was significantly higher than that in the P1 + PBS group or P28 + VEGF group, the number in the P1 + inhibitor group significantly increased compared with that in the P1 + PBS group. ^*^P < 0.05; ^**^P < 0.01; *n* = 6.

As described before, the expression of VEGF was accompanied with the changes of some inflammatory factors in acute inflammation after ST. To further investigate the association of VEGF with inflammation in the local spinal cord below the injury site after ST, we performed immunofluorescence staining in microglia in the L3 segment against Ibal-1 at 8 weeks after injury. The number of microglia in the L3 segment 8 weeks post laminectomy was not significantly different between P1 and P28 sham groups. The accumulation of microglia marked by Ibal-1 immunostaining in neonates and adults was triggered by ST injury compared with that in sham groups (Figure 6A). Administration with VEGF in P28 ST rats significantly attenuated the microglial accumulation compared with that in the P28 + PBS group (Figures 6A5,A6). But after treatment with VEGF, the number of microglia in the P28 ST rats was still higher than that in the P1 + PBS group. Inhibition of VEGF in P1 ST animals significantly increased the number of microglia compared to the P1 + PBS group (Figures 6A,B, *n* = 6, ^*^P < 0.05, ^**^P < 0.01). Hence, VEGF exerts an anti-inflammatory effect during chronic periods after injury.

### VEGF facilitates neuronal survival and neurite growth in LPS-induced motoneuron culture

**Figure 7:**
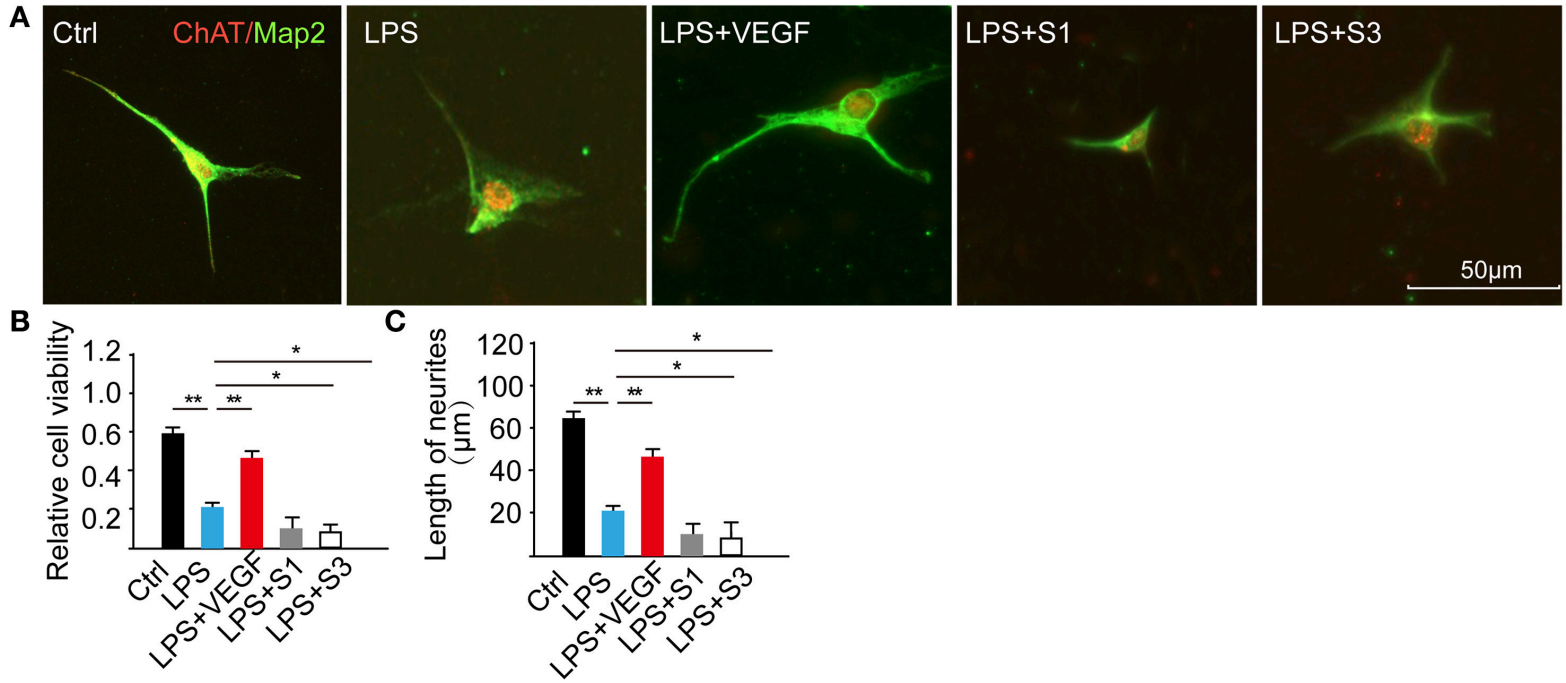
VEGF facilitates neuronal survival and neurite growth. **(A)** E15 cultured spinal motoneurons were immunostained for ChAT (red) and Map2 (green) in five different groups; **(B)** Cell viability was decreased by the treatment of LPS, and the effect was reversed by administration of 0.4 nM VEGF but was intensified following the treatment with VEGF inhibitors: 20 nM S1 and 10 nM S3 (*n* = 20, ^**^the LPS vs. the Ctrl, *P* < 0.01; ^*^P < 0.01; ^**^P < 0.01, the LPS + VEGF, the LPS + S3, the LPS + S1 vs. the LPS, ^*^P < 0.05, ^**^P < 0.01); **(C)** Total neurite length was significantly decreased following LPS-treatment compared with the control (ctrl) but increased in the LPS + VEGF group compared with that in the LPS group; the treatment of inhibitors resulted in shorter neurite length. ^*^P < 0.05; ^**^P < 0.01; *n* = 20.

To determine the direct effect of VEGF on neurons during injuries, we established a LPS-induced inflammation model on E15 cultured spinal motoneurons and analyzed cell viability, neurite out-growth and MAPK signaling pathway. After pretreatment with 0.4 nM VEGF for 2 h, the motoneurons were incubated with 1 μg/ml LPS for 24 h. LPS treatment significantly decreased the cell viability compared with the control, and this effect was partially reversed by administration of 0.4 nM VEGF and diminished by blocking the endogenous VEGF function using VEGFR inhibitors (20 nM S1 and 10 nM S3; Figures 7A,B, ^*^P < 0.05, ^**^P < 0.01). In addition, LPS-treatment inhibited neurite growth compared with the control, and this effect was alleviated by treatment with 0.4 nM VEGF and further worsened by administering VEGF inhibitors (Figure 7C, ^*^P < 0.05, ^**^P < 0.01). Similar to previous reports (Wang et al., 2015), our results indicate that VEGF exerts protection against neuroinflammation and may contribute to functional recovery after injuries.

### VEGF effect is through the MAPK signaling pathway

**Figure 8:**
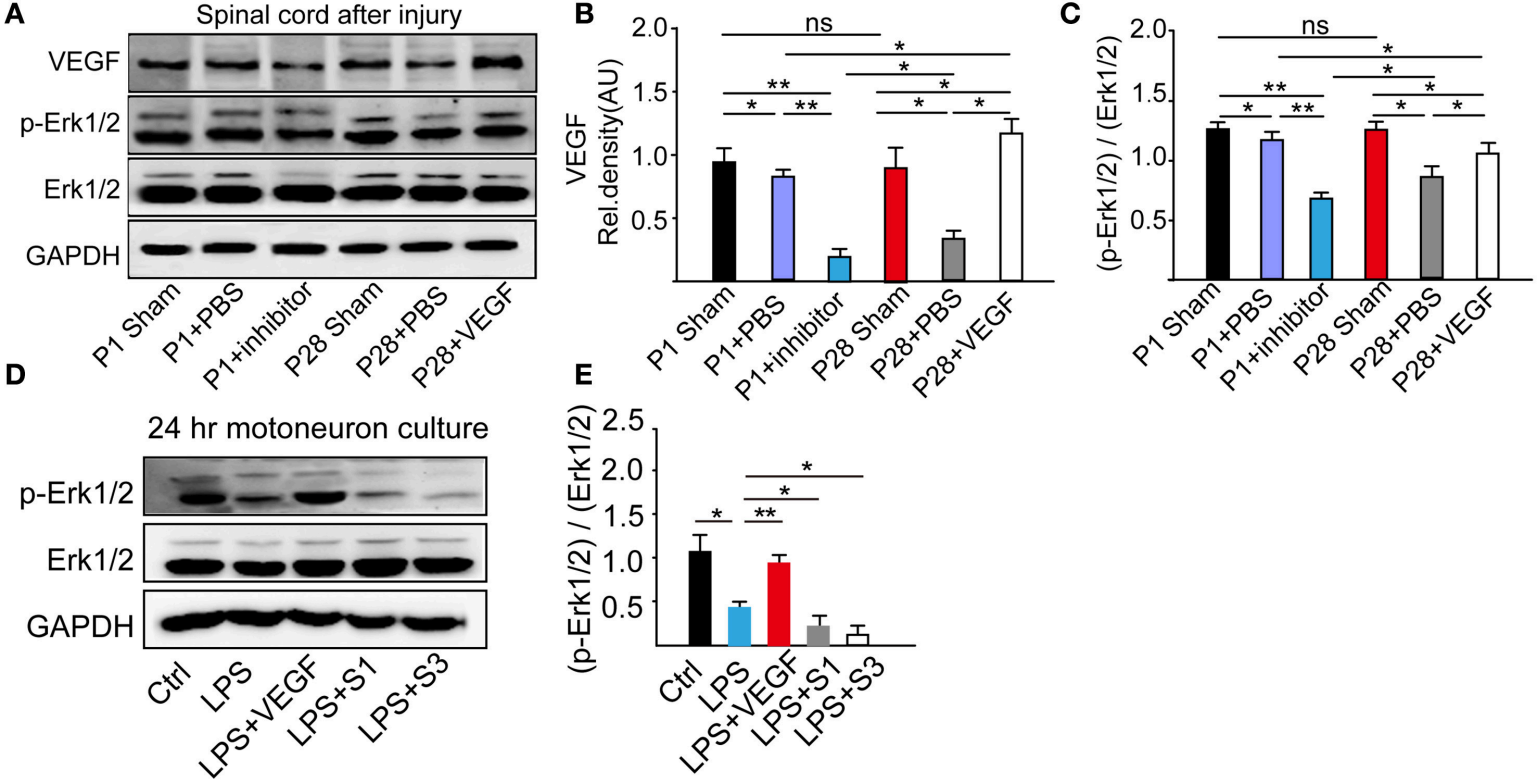
Determination of influence of VEGF on the MAPK signaling pathway. **(A)** Western blots showed the phosphorylation of Erk1/2 from L1–L5 segmental samples 8 weeks after the surgery; **(B)** Quantitative analysis revealed that the VEGF expression reduced after the surgery; treatment of the VEGFR inhibitor down-regulated the VEGF expression level in neonatal animals with ST, and the VEGF administration up-regulated its level in adult rats with injury; **(C)** Phosphorylation levels of Erk1/2 in adults decreased in response to ST, decreased by the inhibition of VEGF in P1 ST rats, but increased by the administration of VEGF in P28 ST animals (*n* = 3 biological replicates, ^*^P < 0.05, ^**^P < 0.01); **(D)** Phosphorylation level of Erk1/2 from 24 h of primary motoneuron culture was assessed by Western blot analysis; **(E)** Western blot quantitative analysis demonstrated that 0.4 nM VEGF can up-regulate the MAPK phosphorylation level, and inhibition of VEGF promoted the reduction MAPK/Erk1/2 phosphorylation level compared with the group treated with LPS (*n* = 20, ^*^P < 0.05, ^**^P < 0.01).

VEGF is a neuroprotective agent that effectively protects both peripheral and central neurons through Erk1/2 and inhibits caspase-3 induction *in vivo* and *in vitro* (Beazley-Long et al., 2013; Xu et al., 2015). Here, to ascertain the role of the MAPK/Erk1/2 pathway in the VEGF effect, we analyzed VEGF expression and Erk1/2 phosphorylation levels through Western blots of L1-L5 samples at 8 weeks after ST (Figure 8A). Administration of VEGFR inhibitor significantly down-regulated the VEGF expression in P1 ST rats compared with that in P1 + PBS and P1 sham groups. In the P28 + PBS group, there were a significant decrease of VEGF expression compared with that in the P28 sham and P1 + PBS groups and a significant increase in the P28 + VEGF group compared with P28 + PBS and P1 + PBS groups (Figure 8B, ^*^P < 0.05, ^**^P < 0.01). Inhibition of VEGF in ST neonatal rats significantly down-regulated the phosphorylation levels of Erk1/2 in contrast to P1 + PBS and P1 sham groups. The phosphorylation levels of Erk1/2 in ST adults significantly reduced in response to ST compared with the P28 sham group. After administration of VEGF, the phosphorylation levels of Erk1/2 in ST adults rats were dramatically up-regulated compared with those in the P28 + PBS group, and did not return to the level in the P1 + PBS group (Figure 8C, ^*^P < 0.05, ^**^P < 0.01). VEGF expression and the phosphorylation levels of Erk1/2 were not significantly different between P1 and P28 sham groups (Figures 8B,C).

From E15 cultured spinal motoneurons pretreated with 0.4 nM VEGF for 2 h and followed with the incubation of 1 μg/ml LPS for 24 h, we found that LPS-treatment decreased the phosphorylation of Erk1/2; the pre-treatment of 0.4 nM VEGF prevented this decreasing; and VEGFR inhibitors further promoted the reduction of LPS-induced Erk1/2 phosphorylation (Figures 8D,E, ^*^P < 0.05, ^**^P < 0.01).

These results demonstrate that VEGF exerts a therapeutic effect on functional recovery in ST rats possibly via the MAPK/Erk1/2 signaling pathway.

## Discussion

Severe SCI leads to neuronal death, axonal loss, demyelination, disrupted neuronal connections between brain and periphery, and corresponding changes in the local microenvironment, eventually resulting in devastating loss of function (Stenudd et al., 2015; Lai et al., 2016). The local environment in the lesioned spinal cord promotes the differentiation of stem cells into glial lineages, including astrocytes and oligodendrocytes (Rossi and Keirstead, 2009; Park et al., 2012). Various extrinsic and intrinsic mechanisms have been established to improve the developmentally related and injury-induced changes that limit plasticity and neuronal survival in the adult CNS (Xia et al., 2014; Gwak et al., 2017). Scholars have reported a large number of molecules that may offer therapeutic strategies for pharmacological inhibition/antagonism. Based on the observations that compared with adult rats, neonatal rats following complete transection had a spontaneous locomotor recovery that may be due to intrinsic plasticity in the lumbosacral circuitry below the lesion controlling hindlimb locomotor activity (Yuan et al., 2013) and a significant difference in the expression of VEGF, the purpose of this study was to investigate the beneficial effects of VEGF on spinal neural survival and circuit reconstruction in lumbar enlargement to improve hindlimb movement in rats undergoing complete T9 transection.

The process of SCI is characterized by initial physical damage (primary injury), leading to progressive damaging processes (secondary injury) spreading away from the injury epicenter (Choi and Rothman, 1990; Patel et al., 2002; Nicola et al., 2017). The primary injury process is predominantly responsible for neuron death, and secondary injuries possess pathological mechanisms involved in inflammatory response, excitotoxicity, ionic imbalance, lipid peroxidation, free radical injury, and apoptosis, which induce neuron death (Yin et al., 2012; Silva et al., 2014). Many studies have investigated various interventions targeting these secondary processes (Beattie, 2004; Jia et al., 2012). Although the expression level of VEGF is relatively low in primary neurons and normal adult brain (Merrill and Oldfield, 2005; Sanchez et al., 2010), it plays essential roles in the CNS undergoing SCI. VEGF is a neurotrophic factor (Choo et al., 2008) that improves the survival of NSCs transplanted into injured spinal cords and enhances angiogenesis in SCI (Oh et al., 2012). The delivery of VEGF and GNDF genes using genetically modified UCB-MCs into the SCI site improves behavioral recovery and axonal regeneration after the injury (Mukhamedshina et al., 2016). As a compensatory response to injury, up-regulation of VEGF occurs in stroke, traumatic injury, aneurysms, and AD and could be directly related to disease pathogenesis (Skirgaudas et al., 1996; Kalaria et al., 1998; Shore et al., 2004; Storkebaum et al., 2004; Merrill and Oldfield, 2005). VEGF also functions as be a modifier of the degeneration of motoneurons in amyotrophic lateral sclerosis (Storkebaum et al., 2004). Moreover, VEGF exerts favorable effects on neuronal survival, which is modulated via the cooperation of neurotrophic, neuroprotective, and angiogenic effects. Previous studies found that nascent mesencephalic neurons show strong and rapid up-regulation of the developmentally regulated structural protein, Map-2, which is coupled with neuritic growth; this finding suggests the role of VEGF in enhanced neuronal maturation (Khaibullina et al., 2004; McCubrey et al., 2006). In the present study, the results revealed that VEGF improved the survival rate of spinal motoneurons, adjusted the structure of motoneurons, and played a functional role in resisting an unconducive environment *in vitro* and *vivo*.

Strong inflammatory responses are a major component of secondary injury after SCI (Jia et al., 2012). Defining the pathways and secondary mediators modulated by the protective candidates is necessary for the development of targeted therapeutics to improve the protective effects. Previous studies indicated that the pathogenesis of chronic neuroinflammatory diseases may contribute to persistent up-regulated levels of VEGF in brain injury or inflammation; hence, VEGF may have a potential effect on anti-inflammation in the CNS (Saban et al., 2008; Wang et al., 2015). In this study, we induced primary rat motoneurons with LPS treatment and performed cytokine profiling of the spinal cord in rats after complete ST. We found that VEGF may exert a therapeutic effect on neural disorders related to neuronal inflammation, such as SCI. *In vivo* studies, the activation of microglia is involved in the pathological condition of SCI and the continuous release of inflammatory mediators accompanied by the influx of macrophages, neutrophils, and lymphocytes (David and Kroner, 2011; Kitayama et al., 2011). Activation of microglia, which induce neuronal cell death and neurite degeneration via Toll-like receptors, promotes the expression and release of pro-inflammatory cytokines to extend and exacerbate the inflammatory responses (Kawabori and Yenari, 2015), leading to functional deficits in the injured spinal cord. Treatments for down-regulating microglial activation are also considered for treatment of SCI (Popovich et al., 1999). Microglial activation during the acute period of SCI can also have a positive effect due to the phagocytosis of the debris and the products of myelin decomposition, contributing to rapid resolution of primary post-traumatic processes. However, microglial activation is unfavorable for neuronal survival in chronic periods after SCI. Our present findings indicated that high VEGF expression regulated the release of inflammatory mediators in acute inflammation, improved the local microenvironment, and inhibited the activation of microglia in chronic periods.

Assessment of neurological function is commonly used measure to evaluate the degree of injury and the therapeutic effect of medications. In the present study, BBB rating and SMEPs indicated significant improvements in locomotion after the treatment. Hence, VEGF may improve the behavioral function in animals after complete ST. Some motor functions can recover, depending on the presence of spinal circuitry that can generate complex sequential activation of various leg muscles after complete spinalization. This is achieved by an intrinsic spinal circuitry, namely, the central pattern generator, which comprises a specialized network of interneurons conducive to generating locomotor patterns, such as alternating activity between flexors and extensors, without supraspinal tracts in the lumbar and sacral spinal cord (Rossignol and Frigon, 2011). The plasticity within intrinsic spinal circuits is a critical component of hindlimb locomotor recovery, and modulating the modified spinal network could contribute to functional recovery in rats that underwent complete ST (Rossignol, 2006). After injury, changes in cellular and circuit properties occur spontaneously and can be mediated through pharmacological, electrical, or rehabilitation strategies (Rossignol and Frigon, 2011). In this study, we illustrated that administration of VEGF enhanced PRV-positive propriospinal connections with spinal motoneurons and remodeled the circuits responsible for hindlimb locomotor recovery in rats after complete ST.

Previous studies have also shown that Erk1/2 signaling cascades exert a key role in the regulation of gene expression and prevention of apoptosis (McCubrey et al., 2006). It is also reported that activation of the MAPK/Erk can promote the differentiation and survival of neurons (Pearson et al., 2001; Yuan et al., 2003). In the present study, VEGF significantly enhanced the survival of spinal cord neurons, mediated the phosphorylation levels of Erk and exerted neuroprotective effects directly through the MAPK/Erk1/2 signaling pathway *in vitro* and *vivo*. Thus, the potential mechanism of VEGF in promoting functional restoration is that VEGF over-expression positively mediates the activity of the MAPK-Erk1/2 signaling pathway, resulting in a reduction in the number of microglia, and a boost in neuronal survival and plasticity of the intrinsic propriospinal circuitry.

In conclusion, this study demonstrated that VEGF regulated spinal inflammatory responses, promoted neuronal survival, enhanced plasticity changes in the intrinsic spinal circuitry, and restored behavioral function in rats after complete ST through the MAPK/Erk1/2 signaling pathways. The results provide a theoretical basis for treatment of patients with spinal cord injury (SCI) and lay a foundation for exploring new targets for therapy.

Our study exhibited several limitations mainly because clinical efficacy, including optimal therapeutic dose and treatment time, could not be determined under animal experimental conditions. Further studies are needed to elucidate the underlying upstream or downstream in MAPK/Erk1/2 signaling molecules that are associated with the protective effects of VEGF in rats after SCI. Overally, VEGF may be a candidate for development of therapeutic agents for SCI.

## Author contributions

WW conceived the study; JL and WW designed the experiment; JL, SC, ZZ, YL, YH, HL, LH, LZ, and WW performed the experiments; JL and WW contributed to data analysis and preparation of figures; JL, LZ, and WW wrote and revised the manuscript.

### Conflict of interest statement

The authors declare that the research was conducted in the absence of any commercial or financial relationships that could be construed as a potential conflict of interest.
