# Supplementary material for: Effect of VEGF on Inflammatory Regulation, Neural Survival, and Functional Improvement in Rats following a Complete Spinal Cord Transection
Source: Front Cell Neurosci. 2017 Nov 29;11:381. doi: 10.3389/fncel.2017.00381 (PMC5712574; doi:10.3389/fncel.2017.00381)
Supplement: Supplementary file 5 [file Table1.DOCX]

Supplementary Table 1. The animals are used in each study.­­­­

|  | P1  Sham | P28 Sham | P1 + PBS | P1 + inhibitor | P28 + PBS | P28 + VEGF | Total |
| --- | --- | --- | --- | --- | --- | --- | --- |
| Behavior test | 12 | 12 | 12 | 12 | 12 | 12 | 72 |
| SMEPs | 12 | 12 | 12 | 12 | 12 | 12 | 72 |
| Nissl | 6 | 6 | 6 | 6 | 6 | 6 | 36 |
| ChAT | 6 | 6 | 6 | 6 | 6 | 6 | 36 |
| PRV tracing | 6 | 6 | 6 | 6 | 6 | 6 | 36 |

SMEPs, spinal motor-evoked potentials; ChAT, cholinergic acetyltransferase; PRV, Pseudorabies virus.
