# Supplementary material for: Effect of VEGF on Inflammatory Regulation, Neural Survival, and Functional Improvement in Rats following a Complete Spinal Cord Transection
Source: Front Cell Neurosci. 2017 Nov 29;11:381. doi: 10.3389/fncel.2017.00381 (PMC5712574; doi:10.3389/fncel.2017.00381)

Supplementary Fig. 1 The expression of some pro- and anti-inflammatory cytokines of P1 and P28 rats following a complete ST. (A) Changes in representative protein expression of anti-inflammatory mediators including IL-10, IL-4 and IL-13 were assessed by the Bio-Plex Pro Rat Cytokine 24-plex Assay. The level of IL-10 was significantly higher in P1 ST rats compared with P28 ST rats at 7 d after injury (n=6, *, $P<0.05$). There was no significant difference in the level of IL-4 between two groups, except for the time point of 12 hr after injury (n=6, *, $P<0.05$). At 6 hr, 12 hr and 7 d after injury, the level of IL-13 in P1 ST rats was lower than that in P28 ST rats (n=6, *, $P<0.05$). (B) Changes in representative protein expression of pro-inflammatory mediators including IL-7, IL-12 and IL-17 were assessed. There were significant changes at every timeline occurred for IL-7, IL-12 and IL-17 between two groups (n=6, *, $P<0.05$).

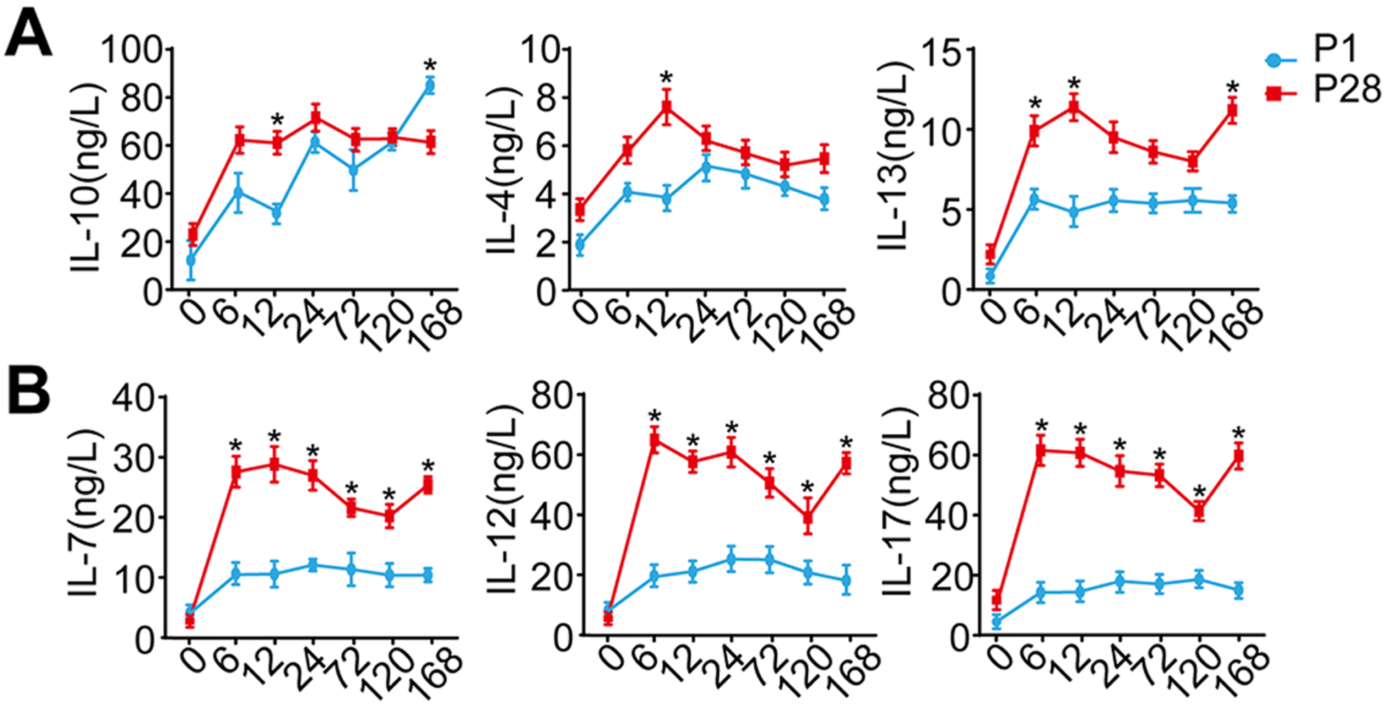

Supplement: Supplementary file 6 [file Image.PDF]
